# Neutralized chimeric DNA probe for the improvement of GC-rich RNA detection specificity on the nanowire field-effect transistor

**DOI:** 10.1038/s41598-019-47522-9

**Published:** 2019-07-30

**Authors:** Wei-Cheng Chou, Wen-Pin Hu, Yuh-Shyong Yang, Hardy Wai-Hong Chan, Wen-Yih Chen

**Affiliations:** 1 Department of Chemical and Materials Engineering, National Central University, Jhong-Li, 32001 Taiwan; 2 Department of Bioinformatics and Medical Engineering, Asia University, Taichung, 41354 Taiwan; 3 Institute of Biological Science and Technology, National Chiao Tung University, Hsinchu, 30010 Taiwan; 4 Helios Bioelectronics, Inc. 3F., No. 2, Sec. 2, Shengyi Rd., Zhubei City, Hsinchu County 302 Taiwan

**Keywords:** Characterization and analytical techniques, Diagnostic devices

## Abstract

Silicon nanowire (SiNW) field-effect transistors (FETs) is a powerful tool in genetic molecule analysis because of their high sensitivity, short detection time, and label-free detection. In nucleic acid detection, GC-rich nucleic acid sequences form self- and cross-dimers and stem-loop structures, which can easily obtain data containing signals from nonspecific DNA binding. The features of GC-rich nucleic acid sequences cause inaccuracies in nucleic acid detection and hinder the development of precision medicine. To improve the inaccurate detection results, we used phosphate-methylated (neutral) nucleotides to synthesize the neutralized chimeric DNA oligomer probe. The probe fragment originated from a primer for the detection of hepatitis C virus (HCV) genotype 3b, and single-mismatched and perfect-matched targets were designed for single nucleotide polymorphisms (SNP) detection on the SiNW FET device. Experimental results revealed that the HCV-3b chimeric neutralized DNA (nDNA) probe exhibited better performance for SNP discrimination in 10 mM bis-tris propane buffer at 25 °C than a regular DNA probe. The SNP discrimination of the nDNA probe could be further improved at 40 °C on the FET device. Consequently, the neutralized chimeric DNA probe could successfully distinguish SNP in the detection of GC-rich target sequences under optimal operating conditions on the SiNW FET device.

## Introduction

Single nucleotide polymorphisms (SNPs) are the most common forms of genetic variations, which are important indicators to disclose individual susceptibility to disease and differences in treatment effect. To achieve the goals of precision medicine, there are strong demands to develop rapid, affordable, easy-to-use, sensitive, and specific techniques for SNP analysis. Researchers have devoted extensive efforts to improve the various techniques for SNP genotyping in the previous decade, such as mass spectroscopy^1^, polymerase chain reaction^2^, microarray^3^, and molecular beacon probes^4,5^. However, most of the abovementioned methods require expensive instruments, complicated procedures, and radioactive/fluorescent labels to amplify the detection signals and sample numbers. Therefore, biosensing techniques have been adopted as platforms of SNP detection due to their high sensitivity, simplicity, short detection time, and good reproducibility. Electrochemical^6^, surface plasmon resonance (SPR)^7–9^ and nanowire-based biosensors are the frequently used platforms for the SNP discrimination^10,11^.

In general, a biosensing platform comprises a recognizing element (the probe) and transducer. Therefore, to enhance the ability of SNP discrimination, the probe design must be enhanced for improved hybridization efficiency and specificity to the target gene molecule. Hence, some DNA analogs, such as peptide nucleic acid (PNA), locked nucleic acid (LNA), and phosphate-methylated (neutral) nucleotide, are applied in the probe design because these analogs have unique properties unfound in nature and can hybridize specifically with natural target DNA. By using LNA modifications on the probe, the melting temperature (T_m_) difference between matched and mismatched duplexes can be adjusted, and a large T_m_ difference may lead to improved performance in mismatch discrimination^12^. Ananthanawat *et al*.^13^ reported that immobilized thiolated-PNA can discriminate between fully complementary DNA from one or two base mismatched DNA on the SPR biosensor. The probe consisting of neutral nucleotides also has been proven to exhibit better DNA detection performance on silicon nanowire (SiNW) field-effect transistor (FET) and SPR biosensors^9,14,15^. The implementation of neutralized chimeric DNA oligomer as a probe enables rapid and selective SNP genotyping during hybridization under low ionic strength and high temperature on the SPR imaging biosensor^9^. For the neutralized chimeric DNA oligomer, the positions of embedded methylated neutral nucleotides in the probe design can influence their performance during hybridization^9,15^. The uncharged properties of these DNA analogs make them attractive to the applications of FET-based biosensors due to their stability and binding capabilities to targets under low ionic strength. However, some sequence limitations in the design of PNA and LNA probes for biosensor application, such as GC content (between 30% and 60%), probe length, and consecutive Gs (less than 3), need to be addressed^16^. Even though the neutralized chimeric DNA oligomer had been proven that it had good performance in SNP genotyping on the SPR based biosensor^9^. There is no definitive proof that such neutralized chimeric DNA oligomers still have excellent genotyping performance on FET-based sensors. Because the sensing mechanisms between SPR and FET biosensors are quite different. SPR is sensitive to changes of the refractive index of the dielectric close to the metal layer of sensing chip. Differ from SPR sensing mechanism, the charges from analytes are the key factor to affect the conductivity of the nanowire channel on the FET-based sensor.

SiNW FET sensors offer ultrasensitive, direct electrical readout, and label-free biological/chemical detection, and they have been applied in the detection of target DNA with low detection limit but high specificity for SNP discrimination^17,18^. Analyte binding influences the density of charge carriers in the channel region, resulting in a change in conductivity. Uncharged probe hybridization with the DNA or RNA target introduces a significant change in charge. By using analogs of DNA probes, the uncharged probe can improve the signal-to-noise ratio of measurement as compared with regular DNA probes and exhibit greater sensitivity than the DNA-modified FET biosensor^19^. Compared with regular DNA probes, our recent study revealed that neutralized chimeric DNAs close to the 5′ end for surface immobilization and alternately modified with phosphate-methylated nucleotides exhibit better sensitivity and specificity in the detection of target fragments^15^. Given the characteristics of SiNW FET devices, variations in the surface charge density close to the NW surface can significantly contribute to the measured signals. In this study, bis-tris propane (BTP) was used to replace conventional mineral buffers in various biological applications. BTP is a zwitterionic buffer with both positive and negative charges and contains trace amounts of metal ions. BTP is a suitable buffer for FET measurements due to its ability to reduce background noise produced by salt ions in buffer and increase the Debye screening length.

**Figure 1:**
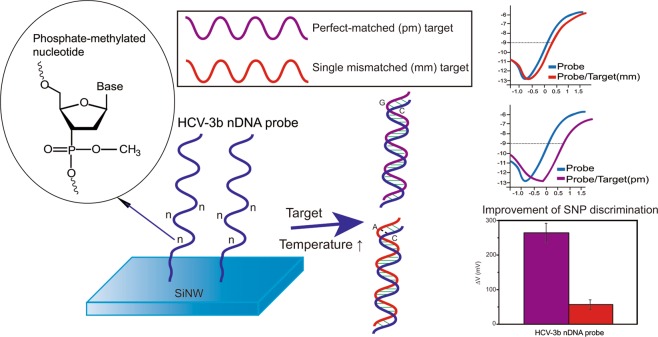
Schematic illustration of the probe and target design and the performance of neutralized chimeric DNA oligomer on the SiNW FET device for SNP detection.

Herein, the target fragment originated from a primer for the detection of hepatitis C virus (HCV) genotype 3b. All oligomers (20-mer length oligos) used in this study are shown in Table 1. HCV is the most common cause of chronic liver disease, and the disease has several genotypes. The probe and target oligomers used were all GC-rich sequences (GC content: 75%), and a high GC content indicated easy formation of secondary structures and high thermal stability. In addition, GC-rich sequences are more difficult to amplify in polymerase chain reaction (PCR), and the natural property of GC-rich sequences can increase the amount of nonspecific binding to produce unstable signals in the application of biosensors. Hence, we adopted phosphate-methylated nucleotides to synthesize partially neutralized chimeric DNA probes by following the rule mentioned in a previous report^15^. The positions of embedded methylated neutral nucleotides must be close to the end of the probe for immobilizing on the sensing surface. These sequences used in this study are different from the reports published previously^9,15^. The major goal of this study is to examine the performance of neutralized chimeric DNA oligomer in the SNP discrimination on the SiNW FET device, especially for the detection of the GC-rich sequence. The influence of hybridization conditions with different concentrations of BTP buffer and temperatures on the performance of the probe for SNP discrimination was also investigated in this study. Eventually, we demonstrate that neutralized chimeric DNA oligomer is able for sensitive SNP genotyping of GC-rich sequence in the low ionic strength buffer and at a higher operating temperature on the SiNW FET device (Fig. 1).

**Table 1:** Sequences of probes and target RNA molecules.

Identifier	Sequence (5′-3′)
HCV-3b probe	NH_2_-C_6_-GCAAGACGCCGCTAGCGCGG
HCV-3b nDNA probe	NH_2_-C_6_-GC^n^AAG^n^ACG^n^CCGCTAGCGCGG
3b-pm	CCGCGCUAGCGGCGUCUUGC
3b-mm	CCACGCUAGCGGCGUCUUGC

Note: phosphate-methylated nucleotides in the sequence were noted by the superscript “n”.

## Material and Methods

### Chemical reagents

Sodium cyanoborohydride, glutaraldehyde (GA), BTP, phosphoric acid, sodium phosphate tribasic dodecahydrate and 3-aminopropyltriethoxysilane (APTES) were brought from Sigma-Aldrich (USA). Tris(hydroxymethyl)aminomethane was purchased from Thermo Fisher Scientific (USA). Acetone and absolute ethanol (99.5%) were acquired from Echo Chemical Co. (Taiwan). BTP buffers were all prepared in deionized water and adjusted the pH to 7.4 by HCl. All other chemicals used in this study were regent grade.

### DNA probes and RNA targets

Hepatitis C virus genome RNA has many genotypes, and the prevalence of HCV genotypes has been well studied. Herein, we adopted a genotype-specific primer that could detect HCV genotyping 3b reported from a literature1 to design the probes and targets used in experiments. Except for HCV-3b nDNA probe, other DNA and microRNA sequences were all synthesized by MDBio, inc. (Taiwan). The chimeric neutralized oligomer (HCV-3b nDNA probe) is also synthesized and supplied by MDBio, inc. (Taiwan), which has phosphate-methylated nucleotides in the backbone as noted by the superscript “n”. One perfect-matched (3b-pm) and one single-mismatched (3b-mm) RNA fragments were used for the hybridization experiments with the probe DNA molecules. The experimental methods of circular dichroism (CD) and SYBR Green for the analysis of oligomers and duplexes and the atomic force microscope (AFM) for checking the morphological characteristics of the chip surface are shown in Supplementary Information.

#### Surface functionalization of poly-Si NWFET sensor

The acquired NWFET chips needed to be immersed in the solution of photoresist stripper for 10 minutes to remove photoresist used in the manufacturing processes. Then, the chips were immersed in deionized water with ultrasonic cleaning for 10 min. After rinsing with de-ionized water, chips were taken out and blown dry with nitrogen gas. An oxygen plasma cleaner (PDC-32G, Harrick Plasma, USA) was used to process the chips for 5 min in order to remove the organic contaminants on the chip surface. After the cleaning process, OH groups presented on the silicon surface and contributed to following surface modifications. Afterward, we immersed the chips in APTES (2 vol% in ethanol) and shook the container filled with nitrogen for 30 min at room temperature. The APTES could link to OH groups and present NH_2_ groups on the surface. The chips were then removed from the container and rinsed thoroughly with ethanol before the 10-min of ultrasonic cleaning. In the ultrasonic cleaning, chips were immersed in ethanol to remove the APTES molecules not attached to the surface. Subsequently, the chips were placed on the heating plate at 120 °C for 10 min. After the heating process, the chips were soaked in the GA solution (12.5 vol% in 10 mM BTP buffer) for shaking 1 h in a dark container. After completing this reaction, a monolayer with the aldehyde (-CHO) terminal group was formed on the NW surface. The FET chips were subsequently cleaned thrice with the BTP buffer and blown dry with nitrogen.

Each HCV-3b or HCV-3b nDNA probe was modified an amino group at the 5′ end of sequence and used to prepare the DNA chip to detect target sequences. We immersed the previously processed chip in the 10 mM BTP buffer containing 1 µM probe DNA (natural or partially neutralized chimeric DNA) and incubated overnight at room temperature. After the overnight immersion, we washed the chip thrice with the BTP buffer and immersed the chip in 4 mM sodium cyanoborohydride (in 10 mM Tris buffer) for 30 min. Cyanoborohydride can react with the immobilized probes and stabilize the C=N groups on the probe backbones. Besides, Tris can quench any unreacted aldehyde sites to reduce the experimental error caused by the nonspecific binding of biomolecule. After reaction, the chip was placed in 10 mM Tris buffer and shaken for 10 min at room temperature. After thoroughly rinsed with deionized water, the chip with immobilized probe molecules was blown dry with nitrogen and stored in a refrigerator for later use.

### NWFET measurements

The poly-Si NWFET sensor used in this study was fabricated by the National Nano Device Laboratories (Hsinchu, Taiwan), which was based on the design of Prof. Yuh-Shyong Yang’s group at the National Chiao Tung University (Hsinchu, Taiwan)^20^. The sidewall spacer formation technique was applied to fabricate the poly-Si NWs of an n-type FET. The detailed descriptions about the fabrication process and characteristics of the poly-Si NWFET sensor were illustrated in previous reports^20–23^. The length and width of each poly-Si NW-channel were 2 μm and 80 nm, respectively. The Keithley 2636 A Dual-Channel System SourceMeter instrument (Tektronix, Inc., USA) was adopted to measure the current-voltage (I–V) characteristics of poly-Si NWFET sensor. A microfluidic system composed of a polydimethylsiloxane flow cell, an acrylic gasket and metal plates, and a programming syringe pump (KD Scientific) and a probe station with a chamber (EverBeing Int’l Corp., Taiwan) were also used in the measurement. Before starting measurements, the prepared chip with immobilized probe molecules was attached to the PDMS flow cell and fixed with clamp. The procedures for the surface functionalization of poly-Si NWFET sensor chip are provided in Supplementary Information. The whole microfluidic system was shown in Fig. S1 and the dimension of fluidic channel was 5 × 0.5 × 0.1 mm^3^.

Basically, the NWFET measurement method used in this study is the same as our previously published study^15^. Initially, the programming syringe pump transported the BTP buffer through the NWFET sensor, and the flow rate was controlled at a constant value of 83 μl/min in all measurements. After incubation for 4 min in the BTP buffer, we started measuring the *I*_d_ − *V*_g_ curves in triplicate at room temperature (25 °C) to ensure the stable signal of the poly-Si NWFET sensor. Until stable signal of the poly-Si NWFET sensor was obtained, we recorded the *I*_d_ − *V*_g_ curve as the baseline to calculate the changes in electric properties caused by the following hybridization events. At that time, the buffer containing target DNA was injected through the chip surface for 10 min. For ensuring better hybridization reactions of the target nucleotide sequences with the immobilized probes, we turned off the syringe pump and incubated for 30 min. In order to remove the non-specific binding of target molecule, the BTP buffer was then pumped into the flow channel for 10 min. We recorded the final *I*_d_ − *V*_g_ curve until the same data was repeatedly measured more than three times.

In order to compare the experimental results quantitatively, the change in gate voltage before and after hybridization was recorded by the quantitative analysis on the data of *I*_d_ − *V*_g_ curves at a drain current (*I*_d_) of 1 × 10^−9^ A as the previously published report^15^. The current magnitude of 1 × 10^−9^ A is approximately the middle of the measured current range, which is a suitable location for the quantitative analysis of gate voltage change. The change in gate voltage before and after hybridization was denoted as ΔV. The procedures of NWFET measurements performed at 40 °C were similar to the experiments carried out at 25 °C. The temperature control of the reaction temperature of 40 °C were done by simply merge the sample in the running buffer in a constant temperature bath and the fluidic system, including all the inlet tubing were rapped with heating tape to maintain the designated temperature.

## Results and Discussion

### Surface functionalization of sensor chip

Considering that the first APTES layer formed on the chip surface is critical to the formation of GA film on APTES and to the results of probe immobilization, we examined the surface morphology of chips in each step of surface modification to determine the optimal condition for the preparation of sensing chips. The alkoxy groups of APTES could directly react with the hydroxyl group on the silica substrate under anhydrous conditions. In hydrous condition, alkoxy groups of silane were subjected to hydrolysis prior to the surface treatment. The amount of water present influences the extent of alkoxy hydrolysis and amine protonation in silane layers^24^. To compare the effect of reaction conditions on film quality, we initially adopted 95% and 99.5% ethanol solutions and selected to fill the container with nitrogen or air. AFM measurements revealed that the purity of ethanol, with or without nitrogen gas treatment in the formation of APTES film, affected the film quality (see Supplementary Fig. S2). The data obtained by multiple measurements (shown in Fig. S3) indicated that uncontrolled polymerization of silane molecules, leading to the possible formation of multilayers on the surface, was effectively avoided by using high-purity ethanol. The APTES films formed in 99.5% ethanol solution demonstrated to have smallest average roughness. The roughness of APTES film with nitrogen gas treatment was the second smallest, but the error bar of roughness was smallest among the four conditions. Hence, we adopted 99.5% ethanol to mix APTES and filled the container with nitrogen gas during film formation on the chip surface.

Silanization of solid surfaces with APTES is sensitive to many factors such as the concentration, reaction time, temperature, moisture, and solvent, all of which affect the quality of SAM. Guha Thakurta and Subramanian^25^ discovered that surfaces silanized with 2 vol% APTES for 30 min could yield a densely populated silane monolayer (1.0–1.2 nm), and this surface could obtain the largest bound density of human immunoglobulin G (HIgG). Our AFM results also revealed that a homogeneous and smooth APTES-modified layer could be obtained by using their methods for the preparation of functionalized FET chips. The uniform APTES-modified layer could achieve high immobilized ligand or protein densities and further improve device performance.

After the formation of the APTES-modified layer, APTES then reacted with a linker molecule, glutaraldehyde, during surface modification. We compared the quality of GA films formed at room temperature and 40 °C by using AFM. Results of probe immobilization on the GA films generated at two temperatures were also examined via AFM. We wanted to observe the surface morphology of chips to determine the conditions that could yield positive results of probe immobilization. The AFM images (shown in Fig. S4a,b) indicated that the quality of APTES-GA film formed at room temperature was better than that of APTES-GA film formed at 40 °C. The chip surface with the APTES-GA film produced at room temperature had an average roughness of 0.3 nm and RMS of 0.67 nm. Even after the immobilization of the DNA probe, the RMS of the APTES-GA-DNA film prepared at room temperature was still relatively smooth compared with that of the film surface formed at 40 °C (shown in Fig. S4c,d). This phenomenon was due to the polymerization of GA in high temperatures^26^.

### The measurements of probe-target duplex formations

**Figure 2:**
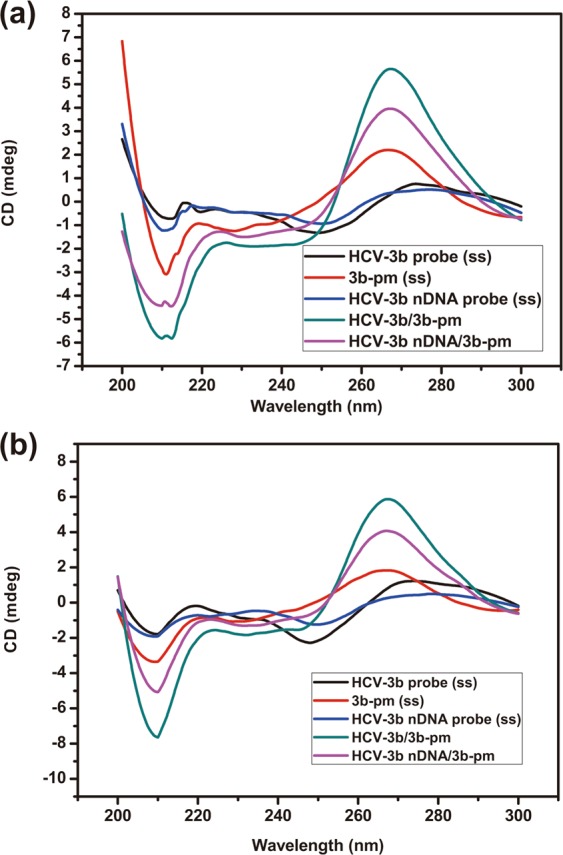
CD spectra of single-stranded probes, the perfect match target (3b-pm), and duplexes in BTP buffers with the concentrations of 100 (**a**) and 10 mM (**b**). The wavelength of the polarized light ranges from 200 nm to 300 nm, and the samples were prepared at 5 μM.

Circular dichroism (CD) is a useful technique for the analysis of DNA, RNA, and protein secondary structures; this technique can also be used to gain information about quadruplex structures of DNA^27^. In this study, the CD spectra were obtained with wavelengths of 200–300 nm, and data were averaged over three scans. DNA samples, including single-stranded probes or perfectly complementary probe-target duplexes, were prepared at 5 μM in BTP buffer solutions with concentrations of 100 and 10 mM. The experimental data obtained in 100 mM BTP buffer are shown in Fig. 2a. On the basis of the characteristic signals of the B-form structure, the spectra should present positive long wavelength bands at around 260–280 nm and negative bands at about 245 nm. The CD spectra of 3b-pm, HCV-3b probe/3b-pm, and HCV-3b nDNA probe/3b-pm duplexes exhibited similar band positions but different band magnitudes. HCV-3b and HCV-3b nDNA probe also could form self-complementary hairpin structures, but the amplitude of CD spectra of two probes at around 260–280 nm was relatively weak. The amount of B-form structure in samples was the major reason for this difference. Moreover, single-stranded 3b-pm presented the more obvious characteristic of a B-form structure, indicating the formation of a self-complementary hairpin structure^9,28^. The transition of duplex to hairpin usually causes a reduction in CD amplitude because of the single-stranded loop^29^. In 10 mM BTP buffer (Fig. 2b), the position and shape of the CD spectra were similar to those in 100 mM BTP buffer. The amplitudes of HCV-3b probe/3b-pm and HCV-3b nDNA probe/3b-pm duplexes were higher than that of single-stranded oligomers. The CD results of our study indicated that buffer concentration and methylated nucleotides on the partially neutralized probe could not significantly influence the formation of secondary structures in the duplex. Similar to previously reported CD results^9^, the effect of ionic strength on the formation of duplex structures for partially neutralized and regular DNA was also not observed in their study.

DNA strands will separate at T_m_, and the temperature depends on several factors: the length of the molecule, sequence, solvent conditions, concentration of nucleic acid, and nucleic acid analog structure^30^. As shown in Table 2, in 100 mM BTP buffer, the melting temperature of HCV-3b nDNA probe/3b-pm (77 °C) was slightly higher than that of HCV-3b probe/3b-pm (76.7 °C). In 10 mM BTP buffer (shown in Table 3), HCV-3b nDNA probe/3b-pm still had a higher melting temperature than HCV-3b probe/3b-pm. These results revealed that the HCV-3b nDNA probe could form a stable helix structure with its complementary target sequence, thereby suggesting that the phosphate-methylated nucleotides on the probe backbone could reduce the electrostatic repulsion force between complementary strands. Other neutral DNA analogs, such as PNA or LNA, also exhibited increments in melting temperature^30–32^. The backbone charge and conformation were important in the improvement of hybridization efficiency and the stability of complex structures. Additionally, the difference in the melting temperatures of the HCV-3b nDNA probe between perfect match and mismatch (ΔT_m_) was slightly smaller than that of the HCV-3b probe whether in 10 or 100 mM BTP buffer. Thus, the HCV-3b nDNA probe demonstrated similar tendency to the regular (HCV-3b) probe under BTP buffer with the same concentration to hybridize the perfect-matched and mismatched targets.

**Table 2:** Melting temperatures of probe/target duplexes in 100 mM BTP buffer.

Identifier	HCV-3b nDNA probe		HCV-3b probe	
	T_m_(°C)	△T_m_(°C)	T_m_(°C)	△T_m_(°C)
3b-pm	77	5.5	76.7	5.7
3b-mm	71.5		71

**Table 3:** Melting temperatures of probe/target duplexes in 10 mM BTP buffer.

Identifier	HCV-3b nDNA probe		HCV-3b probe	
	T_m_(°C)	△T_m_(°C)	T_m_(°C)	△T_m_(°C)
3b-pm	76.4	5.1	75.5	5.4
3b-mm	71.3		70.1	

### Effect of ionic strength and temperature on the detection specificity

**Figure 3:**
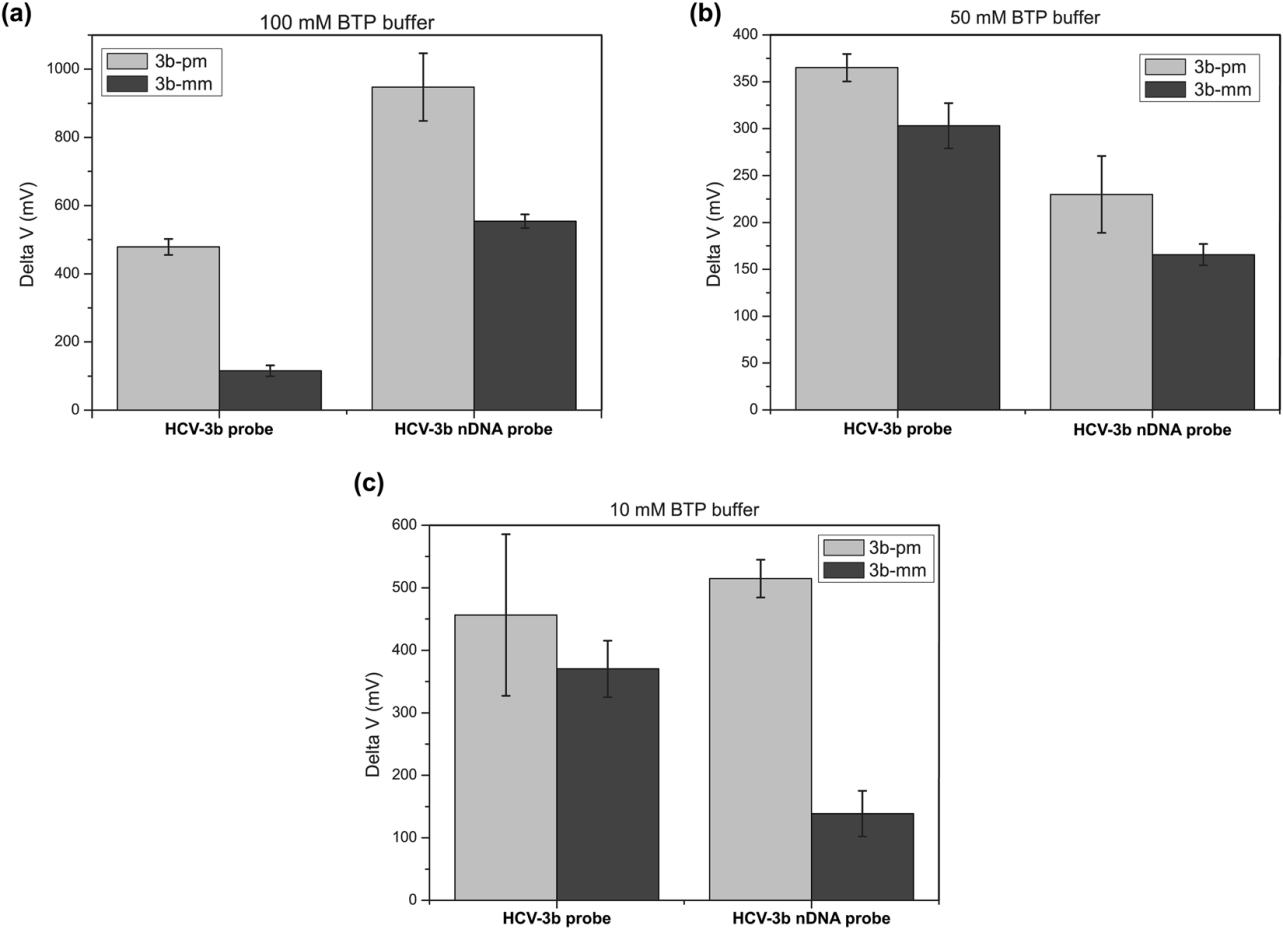
Quantitative data of gate voltage changes produced by probe-target hybridizations in the SiNW FET measurements, which were measured in BTP buffers with the concentrations of 100 (**a**), 50 (**b**), and 10 mM (**c**) at 25 °C.

FETs are highly sensitive biomolecular sensors, which can reach the limit of detection to the femtomolar level^19^ and be applied in SNP detection^18,19,33^. For precision medicine, differentiating SNP genotyping is an indispensable capability of sensing technology. Therefore, we immobilized the HCV-3b and HCV-3b nDNA probes on the sensor surfaces to compare their ability in distinguishing perfect-matched and mismatched targets under different buffer concentrations. These experiments were carried out in BTP buffers with the concentrations of 10, 50, and 100 mM at 25 °C. The perfect-matched and mismatched RNA fragments, 3b-pm and 3b-mm, were all prepared at 1 pM in BTP buffer. The experimental data of each hybridization pairing were obtained from different nanowire devices on the individual chip and recorded with three replicates. The quantitative gate voltage changes of hybridization reactions in 100 mM BTP are presented in Fig. 3a (representative curves are shown in Fig. S5). The HCV-3b probe could interact with the 3b-pm and 3b-mm fragments in 100 mM BTP to produce the average voltage changes of 488.6 and 115.3 mV, respectively. By contrast, the HCV-3b nDNA probe could hybridize with 3b-pm to produce an average voltage change of 947.4 mV and bind to 3b-mm to generate an average voltage change of 554.2 mV. To evaluate the performance of the HCV-3b and HCV-3b nDNA probes on the specificity, the ratio (δ) of voltage change caused by perfectly matched hybridization to that produced by single mismatched hybridization is expressed as follows:

1 $${\rm{\delta }}=\frac{Delta\,voltage\,of\,perfectly\,matched\,sequence}{Delta\,voltage\,of\,single\,nucleotide\,mismatched\,sequence}$$

The δ value reflects the specificity of the probe used to detect the target, and larger δ value also indicates better SNP discrimination. Compared with 3b-mm, 3b-pm could produce larger voltage changes while hybridizing with HCV-3b and HCV-3b nDNA probes. Notably, the δ value for the HCV-3b probe was 4.2, but that for the HCV-3b nDNA probe was only 1.7. The screening of the negatively charged phosphate groups in the backbone of natural probe could reduce electrostatic repulsion with its target fragment by using the buffer with a high concentration. Some studies suggested that unhybridized mismatched nucleotides in the target fragment produce a conformation or energetic barrier to influence the hybridization efficiency in high ionic strength buffer^34,35^. Hence, the signals of HCV-3b probe/3b-pm duplexes were all larger than those of HCV-3b probe/3b-mm duplexes. For the HCV-3b nDNA probe, the neutral backbone of phosphate-methylated nucleotide showed no electrostatic repulsion force between the corresponding nucleotides. Given the reduced electrostatic repulsion force, the hybridization efficiency of HCV-3b nDNA to 3b-pm or 3b-mm increased to produce a significant voltage change during hybridization, resulting in an inferior performance in the specificity of the probe.

Due to the dependence of the Debye screening effect on the ionic strength of the solution, FET devices prefer to be operated in the solution with low ionic strength for getting better detection sensitivity, especially for ultra-low analyte concentration. In real-world and clinically relevant applications on the FET devices, desalting procedures are usually needed to reduce the buffer ionic strength for extending the Debye length. Several Si NWFET nucleic acid sensors had been presented femtomolar or lower limit of detection (LOD) for the detection of DNA hybridization^15,17,36,37^. The HCV-3b probe exhibits a superior performance of specificity in 100 mM BTP buffer. Nevertheless, HCV-3b probe could not have enough detection capability in the detection of analyte with a lower concentration on the FET-based biosensor.

Figure 3b (representative curves are shown in Fig. S6) shows the experimental results performed in 50 mM BTP buffer. The formation of HCV-3b probe/3b-pm and HCV-3b probe/3b-mm duplexes produce average voltage changes of 365 and 303.1 mV, respectively. In addition, the average of voltage change values for the HCV-3b nDNA probe/3b-pm and HCV-3b nDNA probe/3b-mm duplexes were 229.8 and 165.6 mV, respectively. By using Eq. (1), the δ values of the HCV-3b and HCV-3b nDNA probes were 1.2 and 1.4, respectively. At low buffer concentration, less positive molecules could screen the negatively charged phosphate groups of the HCV-3b probe, leading to a large repulsion force during hybridization and interfering with the formation of hydrogen bonds. The barrier for hybridization of the mismatched target to the regular probe may decrease under low buffer concentrations, thereby increasing the levels of nonspecific probe-target hybridization.

The experimental results of probe specificity performed in 10 mM BTP buffer are shown in Fig. 3c (representative curves are shown in Fig. S7). The δ values were 1.2 and 3.7 for the HCV-3b and HCV-3b nDNA probes, respectively. At 10 mM BTP buffer, the δ value of the HCV-3b probe did not differ from that in 50 mM BTP buffer. Notably, the δ value of the HCV-3b nDNA probe significantly increased in 10 mM BTP. This outcome indicated that the nDNA probe could hybridize with its fully complementary target more specifically, and the mismatch target could not hybridize the probe well in this buffer condition. The δ value of the HCV-3b nDNA probe was three times that of the HCV-3b probe, proving that the nDNA probe demonstrated superior specificity under low BTP buffer concentration. In the Figs S5~S7, it is worthy to notice that several current-voltage curves exhibit more than on inflection points, which mainly originates from the device-to-device variation. This device-to-device variation makes it is hard to quantitative determination of the change in the threshold gate voltage. For the quantitative analysis of experimental results, therefore, the drain current at 1 × 10^−9^ A is selected for the measurements of gate voltage changes.

**Figure 4:**
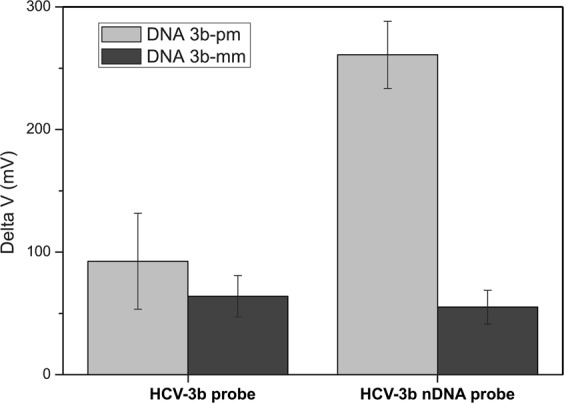
Enhanced SNP discrimination of the probe via SiNW FET measurements in 10 mM BTP buffer and at 40 °C. Quantitative data of gate voltage changes exhibit the operation conditions are benefit for the HCV-3b nDNA probe, and a significant difference is found between the average signals generated by matched and mismatched duplexes.

For further improving the SNP discrimination of HCV-3b nDNA probe in 10 mM BTP buffer, and therefore we anticipated increasing the hybridization temperature to decrease that single mismatch target hybridized with probes. On the basis of T_m_ measured in 10 mM BTP by using the SYBR green PCR assay, the melting temperatures for probe-target duplexes exceeded 70 °C (Table 3). However, the melting temperatures of the duplexes on the solid surface were lower than those measured in the solution^38,39^. For both RNA and DNA, the melting temperatures were lower (∼20 °C) for solid state binding relative to solution^39^. Therefore, we increased the hybridization temperature from 25 °C to 40 °C to lower mismatched hybridization and enhance the specificity of hybridization, resulting in the enhancement of SNP discrimination of the nDNA probe. Ideally, at the hybridization temperature employed, the formation of mismatched duplexes should be affected significantly due to the mismatched duplexes having low melting temperatures. Figure 4 shows the voltage changes produced by the probe-target hybridizations in 10 mM BTP at 40 °C. The experimental results indicated that raising hybridization temperature made a single mismatch fragment difficult to hybridize with the nDNA probe. For the HCV-3b probe, the buffer condition was not conducive to hybridization due to a reduction in charge screening probes and targets, and the measured signal produced by mismatched hybridization at 40 °C also decreased. In Equation (1), the value of SNP discrimination for the HCV-3b nDNA probe reached 4.7 (260.9 mV for 3b-pm; 55.1 mV for 3b-mm), but the discrimination value for the HCV-3b probe was 1.4 (92.5 mV for 3b-pm; 64 mV for 3b-mm). The value of SNP discrimination for the HCV-3b nDNA probe was 3.3-fold to that of the HCV-3b probe. These experiments proved that the discrimination capability of the nDNA probe on the FET devices could be improved by tuning the hybridization temperature and buffer concentration.

Compared with the best performance of regular (HCV-3b) probe in 100 mM BTP, the HCV-3b nDNA probe showed better ability of SNP discrimination in 10 mM BTP at high hybridization temperature. In another study, the neutralized chimeric DNA probe could distinguish perfect-matched and single-mismatched target DNA molecule to the best extent on the SPR biosensor under high temperature and low ionic strength^9^. As DNA analogs, PNA and LNA, using phosphate-methylated nucleotides for probe design could increase the melting temperature of the probe/target complex. The melting temperature of the probe/target complex could be adjusted by altering the number and positions of embedded phosphate-methylated nucleotides in the design of the probe. In particular, the difference in the melting temperatures of the probe between perfect-matched and mismatched targets increases, and specificity can be further improved by increasing the hybridization temperature.

## Conclusions

The present work reports the applications of neutralized chimeric DNA probe for the detection of GC-rich nucleic acid sequences on the SiNW FET device in order to get selective SNP genotyping. The results of CD measurements revealed that the neutralized chimeric DNA probe consisted of phosphate-methylated (neutral) and regular nucleotides could hybridize with the RNA target to form a double-stranded structure. In the viewpoint of the melting temperatures of duplexes, the HCV-3b nDNA probe was more stable than natural DNA probe under 10 mM BTP buffer. At a low concentration buffer (10 mM BTP) and at 25 °C, the detection specificity of the HCV-3b nDNA probe was three times greater than that of the regular DNA probe. After raising the hybridization temperature to 40 °C, the specificity of the HCV-3b nDNA probe further improved, and its δ value was 3.28 times greater than that of regular DNA probe in the same operation condition. Our results indicated that low ionic strength and high operation temperature enabled the HCV-3b nDNA probe to exhibit a large difference in the amount of response between perfect-matched and single-mismatched sequences, resulting in the best performance for SNP genotyping on the poly-Si NWFET sensor. This is the first time we demonstrate that the neutralized chimeric DNA probe can distinguish SNP in the detection of GC-rich target sequences on the SiNW FET device. Based on the results, we can conclude that the main goal of using the neutralized chimeric DNA oligomer in the SNP discrimination for the detection of the GC-rich sequence on the SiNW FET device is achieved, although only one type of mismatched base pair (C-A mismatched) was confirmed in this study. We believe that the neutralized chimeric DNA probe might offer an opportunity for the development of a platform (biosensor or microarray) with high specificity and sensitivity in the detection of GC-rich sequences with appropriate probe design. Nevertheless, the applications of the neutralized chimeric DNA oligomers on the SiNW FET device for the detection of clinical samples, and studying other mismatched base pair combinations still need to be further investigated.

## Supplementary information

Supplementary Information
