# Supplementary material for: Neutralized chimeric DNA probe for the improvement of GC-rich RNA detection specificity on the nanowire field-effect transistor
Source: Sci Rep. 2019 Jul 30;9:11056. doi: 10.1038/s41598-019-47522-9 (PMC6667443; doi:10.1038/s41598-019-47522-9)
Supplement: Supplementary file 1 — Supplementary Information [file 41598_2019_47522_MOESM1_ESM.doc]

**Supplementary Information**

**Neutralized chimeric DNA probe for the improvement of GC-rich RNA detection specificity on the nanowire field-effect transistor**

Wei-Cheng Chou^a^, Wen-Pin Hu^b^, Yuh-Shyong Yang^c^, Hardy Wai-Hong Chan^d^, Wen-Yih Chen^a^,*

^a^ Department of Chemical and Materials Engineering, National Central University, Jhong-Li 32001, Taiwan

^b^ Department of Bioinformatics and Medical Engineering, Asia University, Taichung 41354, Taiwan

^c^ Institute of Biological Science and Technology, National Chiao Tung University, Hsinchu 30010, Taiwan

^d^ Helios Bioelectronics, Inc. 3F., No.2, Sec. 2, Shengyi Rd., Zhubei City, Hsinchu County 302, Taiwan

^*^ Corresponding author: Wen-Yih Chen

TEL:+886-3-4227151 ext.34222

FAX: +886-3-4225258

Email addresses: wychen@ ncu.edu.tw (W.-Y. Chen)

Full postal address: Department of Chemical and Materials Engineering, No. 300, Zhongda Rd., Zhongli District, Taoyuan City 32001, Taiwan

**Materials and Methods**

**Circular dichroism (CD), atomic force microscope (AFM) and SYBR Green**

CD spectroscopy can provide important information about conformational properties of DNA, including A-form, B-form, Z-form, guanine quadruplexes, cytosine quadruplexes, triplexes and other less characterized structures^1^. The most frequently observed conformation of DNA is the B-form, and the base-pairs in the B-form are perpendicular to the double-helix axis. The structures of B-form DNAs are commonly characterized by the CD spectra with a positive long wavelength band or bands at about 260–280 nm and a negative band around 245 nm. The concentrations of single-stranded HCV-3b and HCV-3b nDNA probes used for measuring CD spectra were all 5 μM. The HCV-3b probe/3b-pm and HCV-3b nDNA probe/3b-pm duplexes for the analysis of CD spectra were prepared respectively by mixing 5 μM single-stranded HCV-3b probe or HCV-3b nDNA with its complementary sequences together. The Jasco J810 CD spectrometer was used to record the CD spectra from 200 to 300 nm. The measurement of CD spectroscopy was performed at a constant temperature of 20 °C and data was collected every 0.1 nm interval. The Jasco software equipped with the instrument was applied to smooth the absorbance data by a Fourier transform function. The roughness of the chip surface after chemical modifications was measured by using the AFM instrument (SPA-400 DFM, Seiko, Japan). The AFM instrument was operated in tapping mode to acquire the morphological characterizes of the chip surface.

In addition, melting temperature (T_m_) is an important physical property of nucleic acid duplexes. The melting temperature of the duplex was measured by using SYBR Green quantitative PCR machines. The Applied Biosystems StepOne Plus Real-Time PCR Systems (Thermo Fisher Scientific Inc., USA) could take readings of the amount of double stranded DNA in each well at each PCR cycle. The total volume of prepared sample in each well was 10 μl, which consists of 8.8 μl of bis-tris propane (BTP) buffer, 0.1 μl probe solution (100 μM), 0.1 μl probe solution (100 μM) and 1μl of 5X SYBR Green. In the run method, the reaction conditions were set to heat the temperature to 95 °C for 15 seconds for the denaturation step, and then decrease the temperature to 60 °C in 2°C increments. After reaching 60 °C, the temperature was held for 1 minute. Afterward, the data were recorded by every 0.3 °C increment and kept the temperature for 15 seconds, and the data were stopped recording until the temperature reached 95 °C. Finally, the StepOneTM software v2.3 was used to analyze the data and obtained the melting temperature of the duplex.


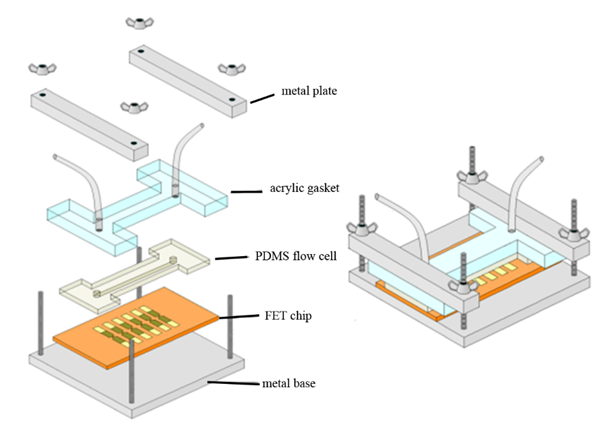


**Fig. S1.** The microfluidic system is mainly composed of a PDMS flow cell, an acrylic gasket and metal plates.

| **95% ethanol** | **95 % ethanol**  **(N_2_ gas)** | **99.5 % ethanol** | **99.5 % ethanol**  **(N_2_ gas)** |
| --- | --- | --- | --- |
| 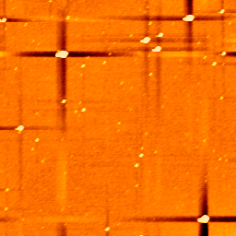 | 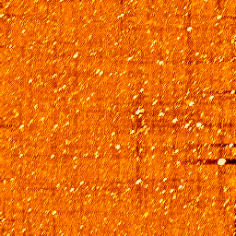 | 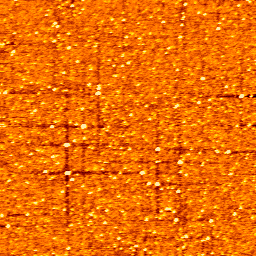 | 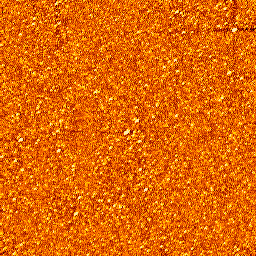 |
| 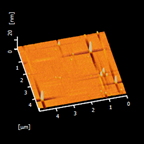 | 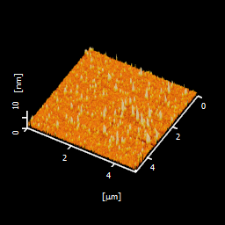 | 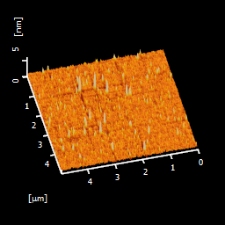 |  |
| Ra: 0.29 nm  RMS: 0.88 nm | Ra: 0.54 nm  RMS: 0.86 nm | Ra: 0.16 nm  RMS: 0.26 nm | Ra: 0.45 nm  RMS: 0.61 nm |

**Fig. S2.** The surface morphology of FET chip surfaces after the modifications of APTES molecule in different conditions (the purity of ethanol, and with or without the treatment of nitrogen gas). The single measurement result for each kind of surface is shown here, and each canning area is 5×5 μm^2^.


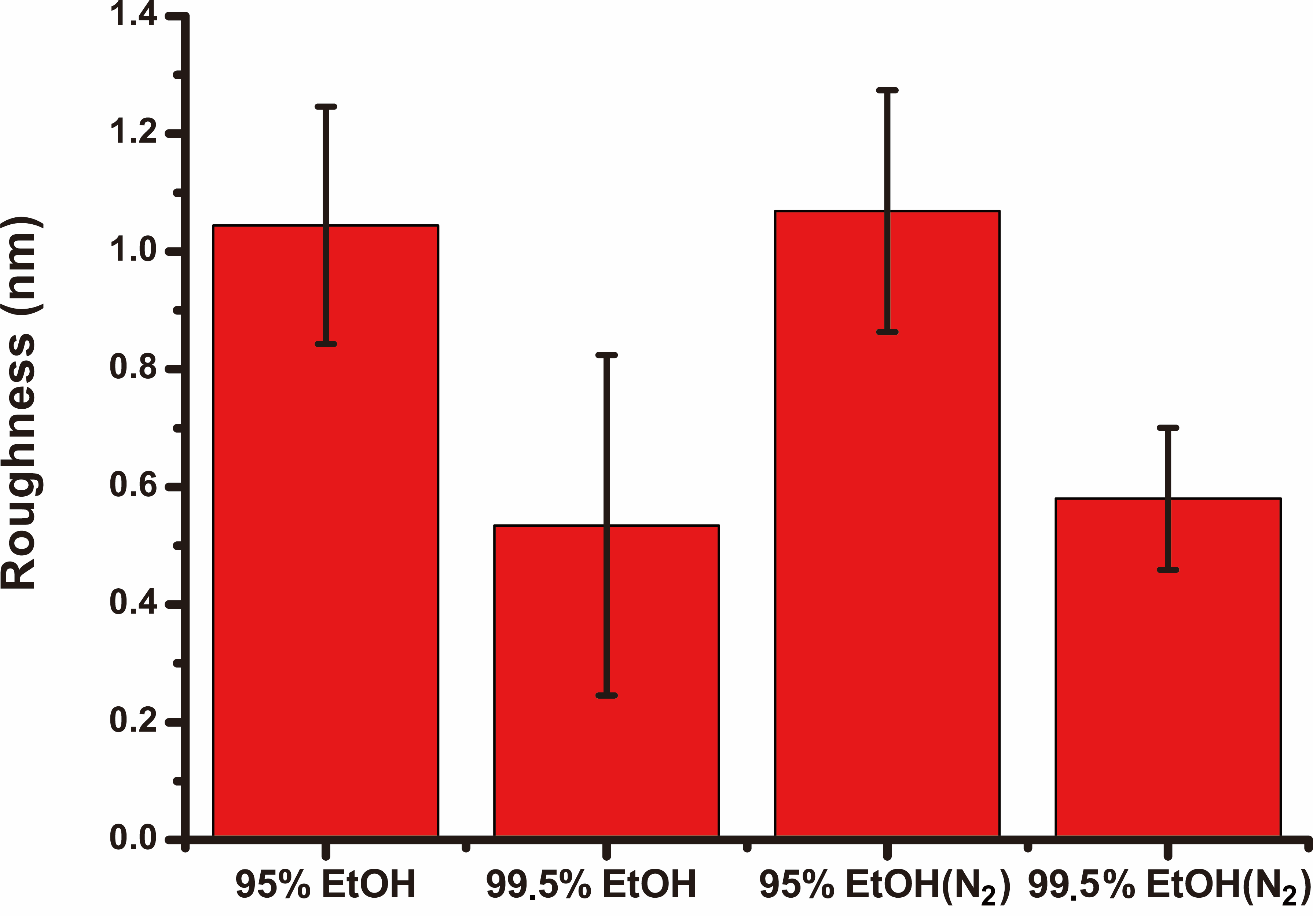


**Fig. S3.** The surface roughness of FET chips obtained from the AFM measurement. The chips with immobilized APTES molecules prepared by using the different purity grades of ethanol, and with or without the treatment of nitrogen gas.

| **The GA film formed at room temperature**  **(a)**  **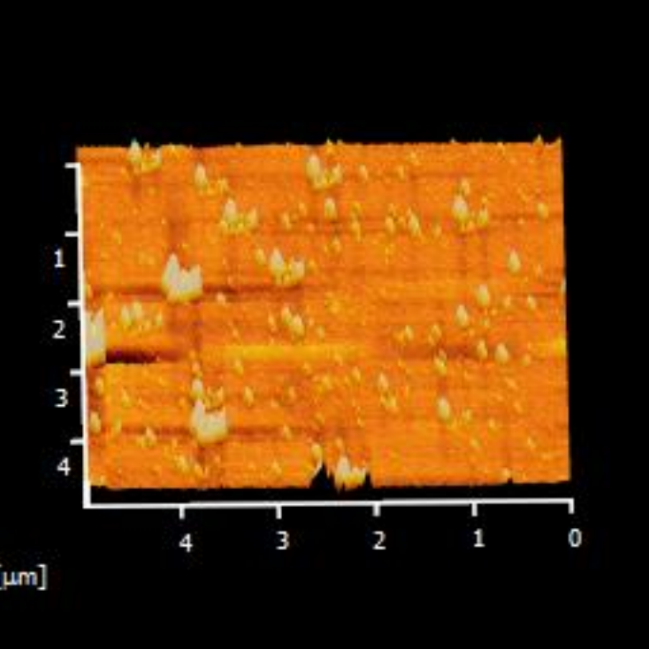** | **The GA film formed at 40 °C**  **(b)**  **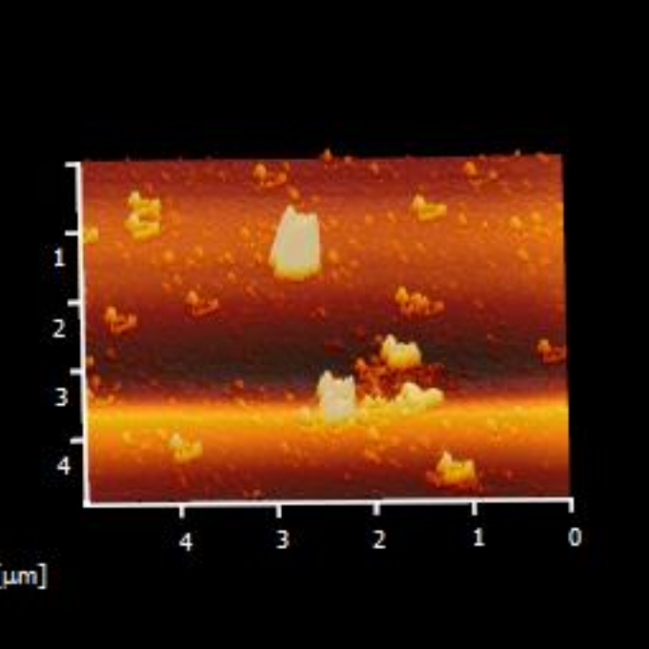** |
| --- | --- |
| **Ra: 0.3 nm**  **RMS: 0.67 nm** | Ra: 1.32 nm  RMS: 2.71 nm |
| **After the immobilization of DNA probe**  **(c)**  **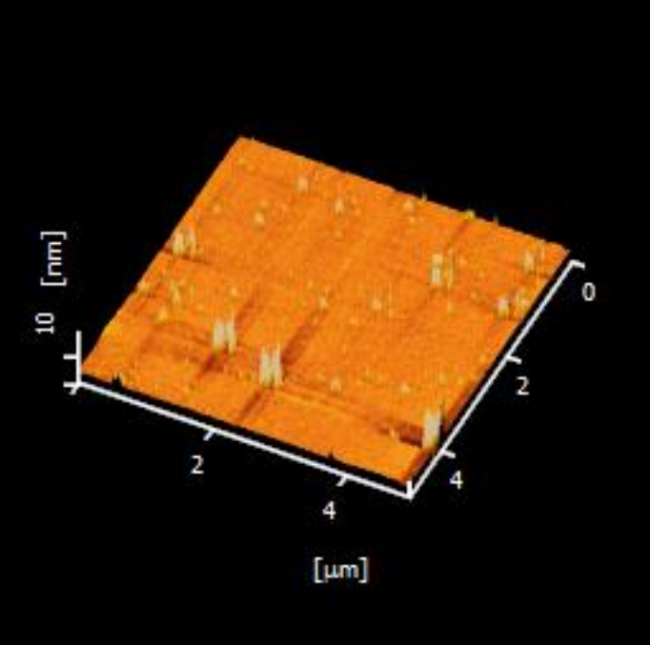** | **After the immobilization of DNA probe**  **(d)**  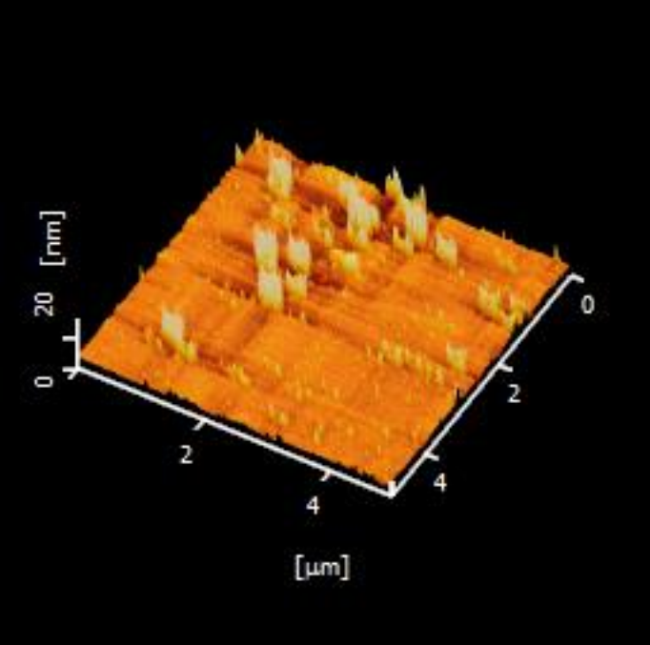 |
| **Ra: 0.61 nm**  **RMS: 1.21 nm** | Ra: 1.28 nm  RMS: 2.3 nm |

**Fig. S4.** AFM images for the FET chips. The GA films were formed at room temperature (a) and 40 °C (b), respectively. (c) and (d) show the surface morphology of chips after the immobilization of DNA probe.

(a)

(b)


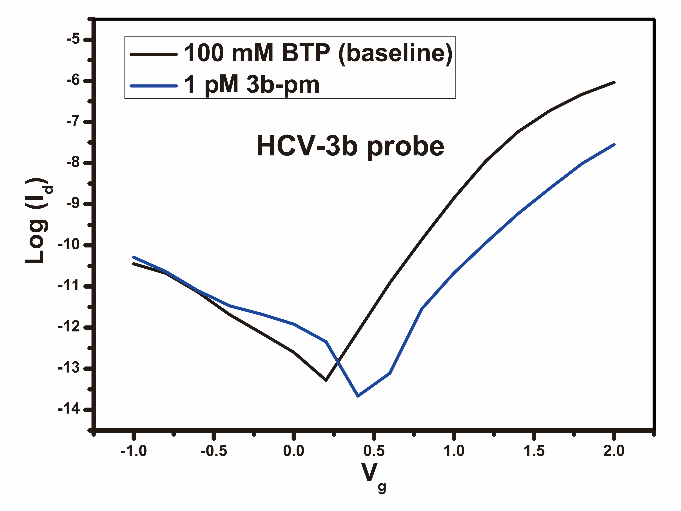

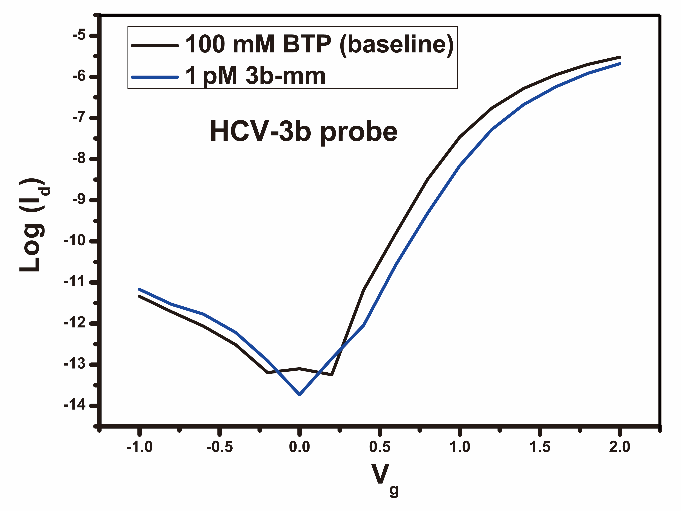


(c)

(d)


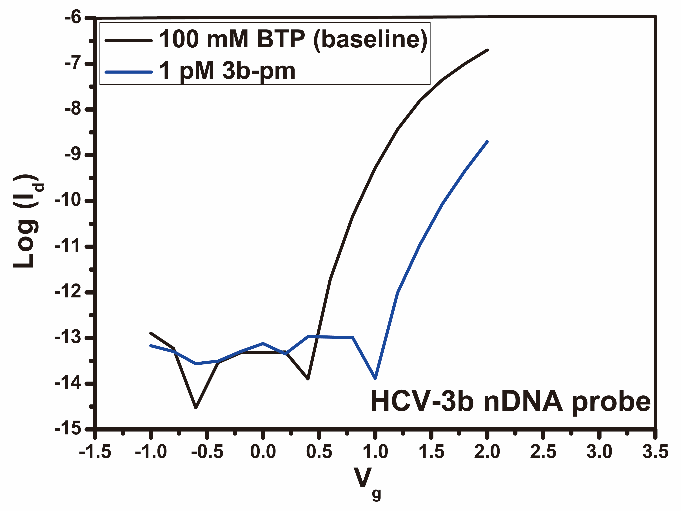

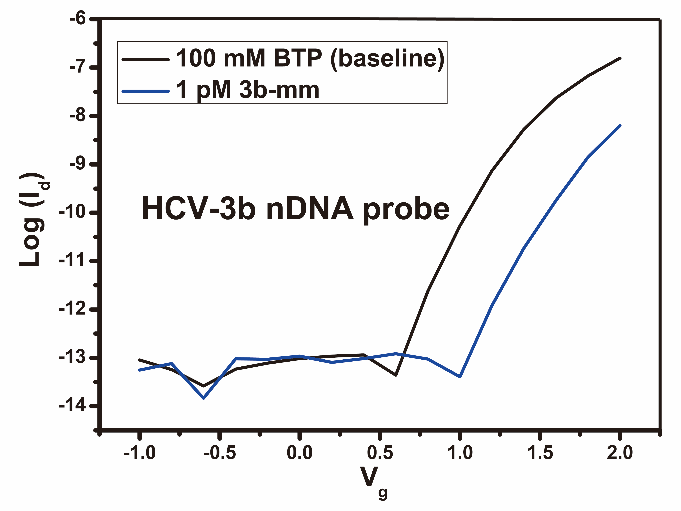


**Fig. S5.** Representative current-voltage curves of probe-target hybridizations measured in the 100 mM BTP buffer by using the FET devices. (a) HCV-3b probe/3b-pm duplex (b) HCV-3b probe/3b-mm duplex (c) HCV-3b nDNA probe/3b-pm duplex (d) HCV-3b nDNA probe/3b-mm duplex

(a)

(b)


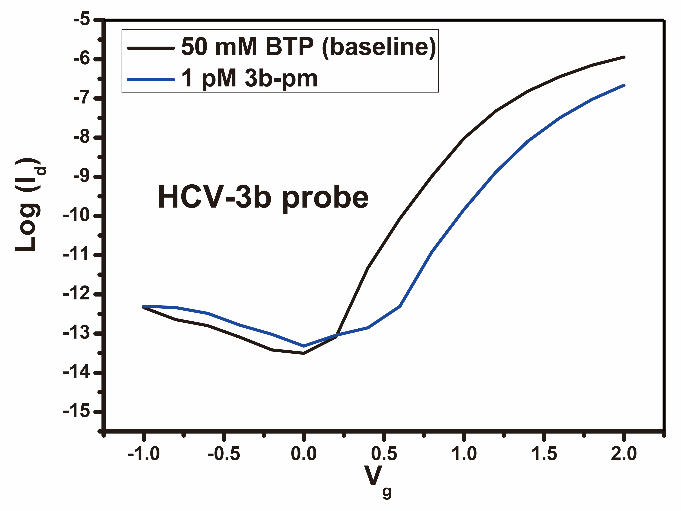

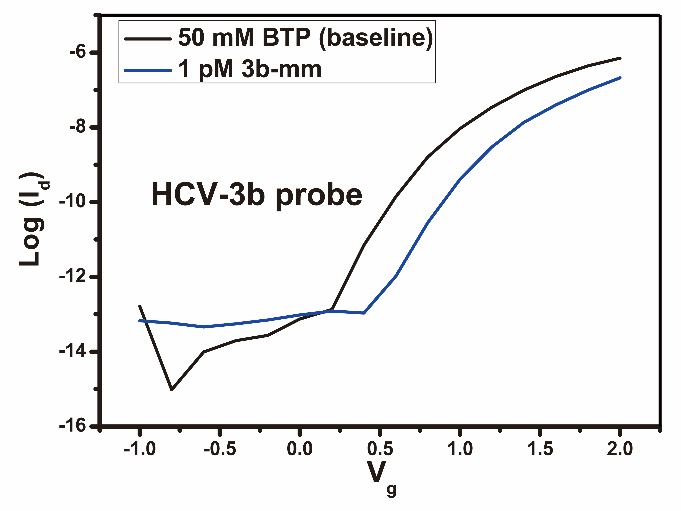


(c)

(d)


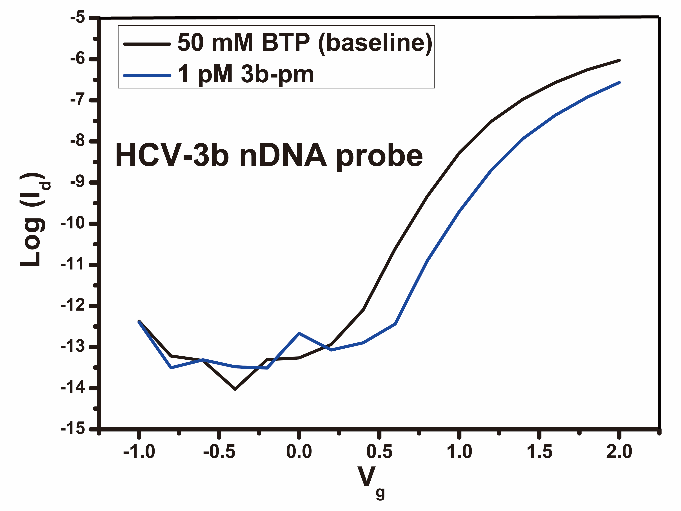

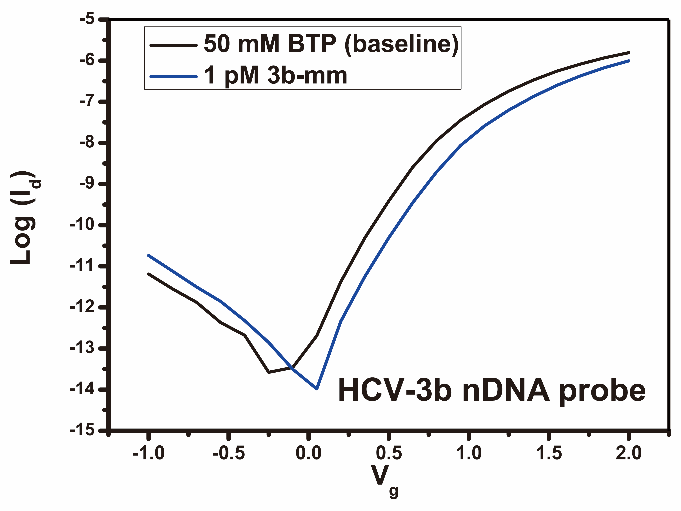


**Fig. S6.** Representative current-voltage curves of probe-target hybridizations measured in the 50 mM BTP buffer by using the FET devices. (a) HCV-3b probe/3b-pm duplex (b) HCV-3b probe/3b-mm duplex (c) HCV-3b nDNA probe/3b-pm duplex (d) HCV-3b nDNA probe/3b-mm duplex

(a)

(b)


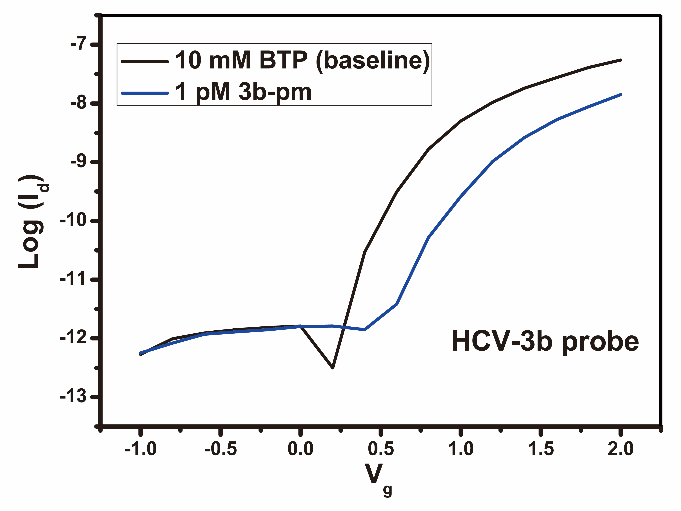

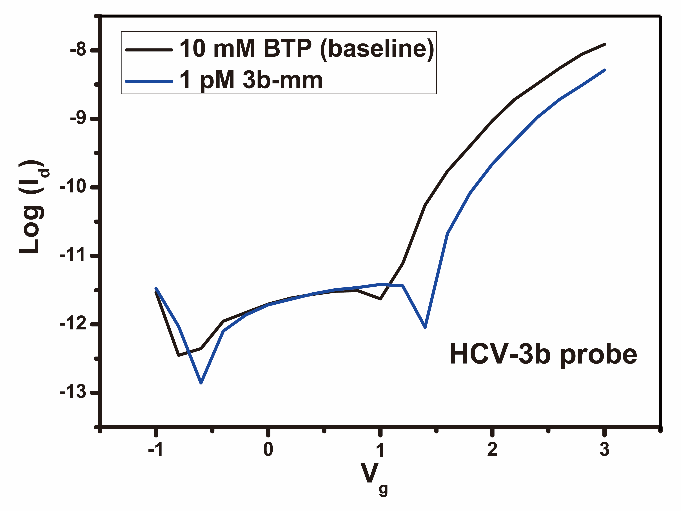


(c)

(d)


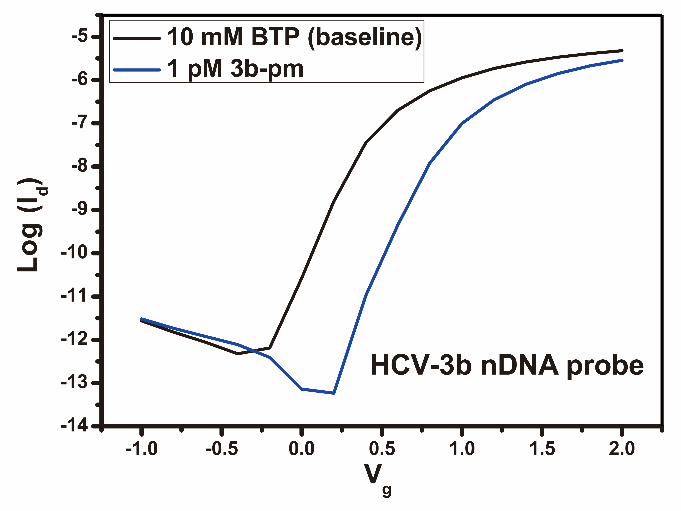

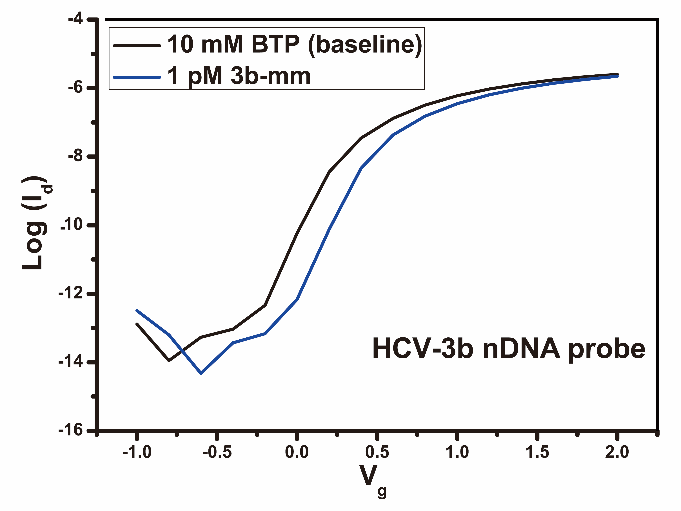


**Fig. S7.** Representative current-voltage curves of probe-target hybridizations measured in the 10 mM BTP buffer by using the FET devices. (a) HCV-3b probe/3b-pm duplex (b) HCV-3b probe/3b-mm duplex (c) HCV-3b nDNA probe/3b-pm duplex (d) HCV-3b nDNA probe/3b-mm duplex

**References**

1. Kypr, J., Kejnovská, I., Renčiuk, D. & Vorlíčková, M. Circular dichroism and conformational polymorphism of DNA*. Nucleic Acids Re*s**. 3**7, 1713–1725 (2009).
